# Identification of Circular RNAs Associated With Chemoresistance in Colorectal Cancer

**DOI:** 10.3389/fgene.2021.696948

**Published:** 2021-09-17

**Authors:** Fei Yao, Xiaochen Xiang, Chuanren Zhou, Qiyou Huang, Xiaoying Huang, Zhufu Xie, Qiang Wang, Qingming Wu

**Affiliations:** ^1^Institute of Infection, Immunology and Tumor Microenvironment, School of Medicine, Wuhan University of Science and Technology, Wuhan, China; ^2^Hubei Province Key Laboratory of Occupational Hazard Identification and Control, Wuhan University of Science and Technology, Wuhan, China

**Keywords:** circRNA, colorectal cancer, chemo-resistance, RNA sequencing, hsacirc_002482

## Abstract

Chemoresistance is a major clinical obstacle for the treatment of colorectal cancer (CRC). Circular RNAs (circRNAs) are a new type of non-coding RNA that participated in the development of chemoresistance. However, the profiles and effects of circRNAs in 5-fluorouracil (5-Fu) and cisplatin resistance of CRC are still unclear and need to be elucidated. In the present study, the profiles of circRNAs in CRC chemoresistant (HCT8/5-Fu and HCT8/DDP) and chemosensitive (HCT8) cell lines were identified *via* RNA-sequencing. In total, 48 and 90 differentially expressed (DE)-circRNAs were detected in HCT8/5-Fu and HCT8/DDP cell lines, respectively. Gene Ontology enrichment and Kyoto Encyclopedia of Genes and Genomes pathway analysis were conducted on the host genes of DE-circRNAs; the results showed that the most significant enrichment pathways in HCT8/5-Fu and HCT8/DDP cell lines were base excision repair and Hippo signaling pathway, respectively. In addition, 11 common DE-circRNAs in the two drug-resistant cell lines (two are upregulated and nine are downregulated) were screened and verified by quantitative real-time PCR; hsacirc_023607 and hsacirc_007420 were found to be the circRNAs with the highest upregulation and downregulation fold changes. However, functional studies showed hsacirc_023607 has no effect on CRC chemoresistance. Therefore, the regulatory networks of targeted miRNAs related to 5-Fu or cisplatin resistance were predicted and constructed, in which hsacirc_002482 was identified as a hub gene, and its overexpression could suppress HCT8/5-Fu and HCT8/DDP cell proliferation and promote cell apoptosis, and enhance cell chemosensitivity. Taken together, these results of the study suggested that hsacirc_002482 may play important roles in chemoresistance of CRC.

## Introduction

Colorectal cancer (CRC) is a common malignant tumor of the digestive system, with high morbidity and mortality (Bray et al., 2018). Surgical resection is still the main treatment for CRC at present, but for inoperable or advanced patients, chemotherapy becomes an important therapeutic approach (Aakif et al., 2016). 5-Fluorouracil (5-Fu) combined with cisplatin (DDP) can reduce the recurrence rates and prolong survival time of patients (Martini et al., 2017). However, the patients usually acquired chemoresistance to chemotherapeutic drugs after a period of treatment, resulting a poor prognosis (Amable, 2016; Ghosh, 2019). Thus, a deeper understanding and exploration of mechanisms of chemoresistance are crucial to improve the efficacy of chemotherapy in CRC.

Circular RNAs (circRNAs) are a category of non-coding RNA with a covalently closed loop structure, mainly formed by introns or exons through back-splicing or lariat introns, which could function as cancer biomarkers because its circular structure was more stable and not susceptible to degradation by RNA exonuclease (Kristensen et al., 2019; Patop et al., 2019). CircRNAs were regarded as error products of spliceosome-mediated and have not been further studied until recent years (Sanger et al., 1976; Li et al., 2020). The development of high-throughput technology provides the possibility for in-depth study of circRNAs. Accumulating evidence demonstrated that circRNAs were not only involved in numerous biological processes (BPs), such as cell proliferation, apoptosis, invasion, and migration but also related to the chemoresistance of various cancers (Salzman, 2016; Jeyaraman et al., 2019; Wang et al., 2019). For example, Hon et al. (2019) indicated that has_circ_0000338 was highly expressed in FOLFOX-resistant HCT116 cells compared with parental cells, and knockdown has_circ_0000338 could improve the chemosensitivity. Besides, it was found that circRNA-SORE was highly expressed in sorafenib-resistant hepatocellular carcinoma cells. Mechanically, circRNA-SORE could bind with the oncogenic protein YBX1 in the cytoplasm, preventing YBX1 degradation mediated by PRP19; sorafenib resistance was overcame when silencing circRNA-SORE (Xu et al., 2020). These studies indicated circRNAs may play important regulatory roles in cancer chemoresistance. However, the expression profiles and effects of circRNAs in 5-Fu and cisplatin resistance of CRC are still unclear and need to be elucidated.

In the present study, to further explore the relationship between circRNAs and chemoresistance of CRC, we first detected circRNA expression profiles and screened the differentially expressed (DE) circRNAs in two different drug-resistant cell lines (HCT8/5-Fu and HCT8/DDP) compared to CRC parental cells (HCT8) through RNA-sequencing, and performed Gene Ontology (GO) and Kyoto Encyclopedia of Genes and Genomes (KEGG) pathway analysis. In addition, to further find circRNAs both involved in 5-Fu and cisplatin-resistance of CRC, 11 common DE-circRNAs in the two drug-resistant cell lines were screen out and further verified by quantitative real-time PCR (qRT-PCR), among which hsacirc_023607 was the circRNA with the highest upregulation. However, we found that silencing hsacirc_023607 does not affect CRC chemoresistance, indicating not all common DE-circRNAs related to drug resistance; further research is needed. Thus, the targeted miRNAs associated with 5-Fu or cisplatin resistance of the common DE-circRNAs were predicted, and the circRNA–miRNA regulatory networks were constructed. hsacirc_002482 was identified as a hub gene, which was decreased in two chemoresistance cells. Gain-of-function assays showed that hsacirc_002482 overexpression suppressed HCT8/5-Fu and HCT8/DDP cell proliferation, promoted cell apoptosis and enhanced cell chemosensitivity. Taken together, these results suggested that hsacirc_002482 may play important roles in chemoresistance of CRC.

## Materials and Methods

### Cell Culture

The human CRC cell line HCT8 was purchased from China Center for Type Culture Collection (Wuhan, China). The parental cells HCT8 were exposed to increasing concentrations of 5-Fu or cisplatin for more than 7 months to obtain the chemoresistant CRC cell lines (HCT8/5-Fu and HCT8/DDP). Both cells were cultured in RPMI-1640 (Gibco, Carlsbad, CA, United States) supplemented with 10% fetal bovine serum (Gibco, Carlsbad, CA, United States) and 1% penicillin/streptomycin (Gibco, Carlsbad, CA, United States). In addition, HCT8/5-Fu cells were cultured with 5 μg/ml 5-Fu (Sigma-Aldrich, St. Louis, MO, United States), and HCT8/DDP cells were cultured with 1 μg/ml cisplatin (Sigma-Aldrich, St. Louis, MO, United States) to maintain drug resistance at 37°C in a 5% CO_2_ incubator.

### MTT Assay

The cells in logarithmic growth phase were seeded in a 96-well plate with 5,000 cells per well for overnight incubation, and five duplicate wells were set for each group. Different concentration gradients of 5-Fu or cisplatin were added into wells for 48 h to assess cell viability. Same-concentration gradients of 5-Fu or cisplatin were supplemented into wells and cultured for 12, 24, and 36 h to evaluate cell proliferation ability. MTT reagent (100 μl; Sigma, United States) was added into each well and then incubated for 4 h at 37°C. After that, 150 μl of dimethyl sulfoxide (DMSO) was added, and the absorbance was detected at 490 nm with a spectrophotometer.

### RNA Extraction and Quality Assessment

The parental cells and drug-resistant cells were placed at room temperature for 5 min for fully lysis after adding TRIzol reagent (Invitrogen, Grand Island, NY, United States). Chloroform was added according to 200 μl chloroform/ml TRIzol, and then shook and mixed well. All samples were centrifuged at 12,000 × *g* for 15 min at 4°C. The upper water phase was taken and transferred into another centrifuge tube, and then 0.5 ml isopropanol was added and placed at 4°C for 10 min. The RNAs were at the bottom of the tubes after centrifugation and the supernatant was discarded, then they were washed and suspended in 75% ethanol, centrifuged, and dried at room temperature for 5–10 min; finally, DEPC water was added to dissolve it. The NanoDrop 2000 spectrophotometer (Thermo Scientific, Rockford, IL, United States) was used to perform preliminary quantification of RNA, and agarose gel electrophoresis and Agilent 2100 (Agilent Technologies, Santa Clara, CA, United States) were used to evaluate RNA integrity; the purity of the RNA was evaluated *via* the ratio of OD_260_/OD_280_. The RNA integrity number (RIN) value is a quantitative value reflecting the integrity of RNA. In general, the RNA samples were used for further experiments with RIN ≥ 7 and OD_260_/OD_280_ ratio between 1.8 and 2.1.

### RNA-Sequencing

After extracting the total RNAs of the parental cell and the two drug-resistant cells, the Ribo-Zero Magnetic kit (Epicentre, Madison, WI, United States) was used to remove the rRNA from the total RNA, and then RNase R (Epicentre, Madison, WI, United States) was used to remove linear RNAs and enrich circRNAs. The remaining RNAs were interrupted to fragments about 300 base pairs (bp) in length. The first-stranded complementary DNA (cDNA) was synthesized with random hexamer primers, and the second-strand of cDNA was digested with USER Enzyme (NEB, Ipswich, United States) before PCR amplification. Subsequently, the quality, total concentration, and effective concentration of the library were detected by the Agilent 2100 Bioanalyzer (Agilent Technologies, Santa Clara, CA, United States), and QuantiFluor dsDNA System (Promega, Madison, WI, United States) was used to quantify libraries. The libraries were sequenced based on the Illumina HiSeq 2000 platform, and 150-bp paired-end reads were generated. There were three replicates in each group, and all sequencing was completed by Majorbio Biotech Co., Ltd. (Shanghai, China).

### Identification of DE-Circular RNAs

Raw data were filtered to obtain high-quality sequences; HISAT2^1^ was used to map clean data to the reference genome, and then reads aligned to the genome were performed comparison regional distribution and gene coverage uniformity analysis. Find_circ tools were used to identify the circRNAs (Jeck et al., 2013). The expression levels of each transcript were quantified by the reads per kilobase of model per million base pairs sequenced (RPKM). The analysis of differences in circRNAs expression between the two groups was performed using the DESeq software (Hansen et al., 2016). The DE-circRNAs were selected with |log2FoldChange| > 1 and *p* < 0.05. To further find circRNAs involved in 5-Fu and cisplatin resistance in CRC, the common DE-circRNAs were selected between the two drug resistance groups.

### Gene Ontology and Kyoto Encyclopedia of Genes and Genomes Enrichment Analysis

Gene Ontology^2^ and KEGG^3^ analyses of DE-circRNAs were performed to determine the main biological functions and significantly enriched pathways. *p* < 0.05 was considered statistically significant.

### Quantitative Real-Time PCR

To verify the accuracy of the sequencing results, the expression levels of common DE-circRNAs in two drug-resistant cells were detected by qRT-PCR. Total RNA was extracted from drug-resistant cells by using TRIzol reagent (Invitrogen, Grand Island, NY, United States), and complementary DNA (cDNA) was synthesized using the ReverTra Ace qPCR RT Kit (TOYOBO, Japan) according to the instructions. Subsequently, quantitative PCR was conducted using iTaq^TM^ Universal SYBR Green Supermix (Bio-Rad, Hercules, CA, United States). The primer sequences of common DE-circRNAs used in the study were synthesized by Sangon Biotech (Shanghai, China) and shown in Table 1. GAPDH was used as the internal control, and the relative expression of circRNA was calculated by the 2^–ΔΔ*Ct*^ method. Each sample was analyzed in triplicate.

**TABLE 1:** Sequences of the primers in the analysis of circRNA expression by qRT-PCR.

Name	Primer sequence	Product (bp)
GAPDH	Forward: 5′-CCAGCAAGAGCACAAGAGGAAGAG-3′	109
	Reverse: 5′-GGTCTACATGGCAACTGTGAGGAG-3′	
hsacirc_030252	Forward: 5′-GGGACACATTCTGGCTCATGC-3′	144
	Reverse: 5′-CGCCACAACTTGATCCTCCTTC-3′	
hsacirc_027876	Forward: 5′-CACCCACAGCGCCTATCTCA-3′	116
	Reverse: 5′-ACTCTGGGCTTCACTGGTGC-3′	
hsacirc_023607	Forward: 5′-GGCTCTGGCGTTGGTGTTTT-3′	133
	Reverse: 5′-CGTTGGCTGCCATCACTGTC-3′	
hsacirc_018467	Forward: 5′-AGAAAAAGAGCAAGAGGCCATTTCT-3′	109
	Reverse: 5′-AGTGGTCACGGTCCAGTACA-3′	
hsacirc_016764	Forward: 5′-GCTCTCCTTGCACCTGATCAA-3′	114
	Reverse: 5′-TTGTGATGTAAAACAGGAAGCAAGG-3′	
hsacirc_016305	Forward: 5′-AGGCATCTCAAGAGACTTGCGT-3′	93
	Reverse: 5′-TGGGCATCCAGAAGTGGGTC-3′	
hsacirc_008249	Forward: 5′-TACGCCATGGAAACCGCTCT-3′	80
	Reverse: 5′-TCCGCTGGTAATCCCCATCG-3′	
hsacirc_007420	Forward: 5′-CCACCAGACGAGCACCAAGA-3′	125
	Reverse: 5′-GAGTGCAGTGAAGCGTTCGG-3′	
hsacirc_006554	Forward: 5′-TGGCTGGTTTCCTGGACAGA-3′	104
	Reverse: 5′-TGCCTTCAGGATAGCGCTCT-3′	
hsacirc_002482	Forward: 5′-AAGCTAAAACCATGGGGGCAA-3′	138
	Reverse: 5′-CCTTCTGAAGGTACCTTTGAATCTCT-3′	
hsacirc_000154	Forward: 5′-GAGATGTGGACCGTGTGAAAAGA-3′	150
	Reverse: 5′-TCAAGGACTCAGAGAGCCGT-3′	

### Prediction of Circular RNA–miRNA Networks

miRanda and circinteractome database were used to predict the targeted miRNAs of common DE-circRNAs. The circRNA–miRNA networks were displayed, and the hub gene was identified by Cytoscape software.

### Cell Transfection

hsacirc_002482 overexpression plasmids (hsacirc_002482-OE) and its negative control were constructed and purchased from GenePharma (Shanghai, China). When the cell confluence reached 50%, transfection was performed using Lipofectamine 2000 reagent (Invitrogen, CA, United States) according to the instructions of the manufacturer.

### Western Blotting

Proteins were lysed by radioimmunoprecipitation assay (RIPA) lysis buffer with phosphatase inhibitor (Beyotime Biotechnology, Shanghai, China), and protein concentrations were quantified using a bicinchoninic acid (BCA) Protein Assay Kit (Biosharp, Shanghai, China). Protein samples were separated by 8.75% SDS-PAGE gel and transferred to a polyvinylidene fluoride (PVDF) membrane (Bio-Rad, United States), and then the membrane was blocked in 5% skim milk at room temperature and incubated with primary antibodies for γH2AX (1:1,000, ABclone, AP0099) and β-actin (1:5,000, ABclone, AC026) overnight at 4°C. Membranes were incubated with horseradish peroxidase (HRP)-conjugated secondary antibodies at room temperature for 1 h, and then visualized with ECL reagents (Bio-Rad, United States). The relative level of γH2AX protein expression was determined by densitometric analysis using ImageJ software.

### Apoptosis Assay

The chemoresistance cells transfected with overexpression plasmid were seeded into six-well plates, and then 5-Fu and cisplatin were added into HCT8/5-Fu and HCT8/DDP cells for 48 h, respectively. The cells were collected and washed with precold PBS, and then about 1–5 × 10^5^ cells were resuspended in 500 μl of binding buffer with 5 μl of annexin V–fluorescein isothiocyanate (FITC) and 10 μl propidium iodide (PI) staining solution for 15 min at room temperature. Accuri C6 (BD Biosciences, Franklin Lakes, NJ, United States) flow cytometer was used to measure the cell apoptosis, and data were analyzed by FlowJo software (Tree Star, Ashland, OR, United States).

### Statistical Analysis

SPSS 25.0 statistical software was used for data analysis. The experimental data were expressed as mean ± SD. The independent sample *t*-test was used for the comparison of means between the two groups, and *p* < 0.05 was considered statistically significant. The expression difference of circRNAs was considered to be statistically significant with |log2FoldChange| > 1 and *p* < 0.05.

## Results

### Identification of Colorectal Cancer Chemoresistant Cell Lines

**FIGURE 1:**
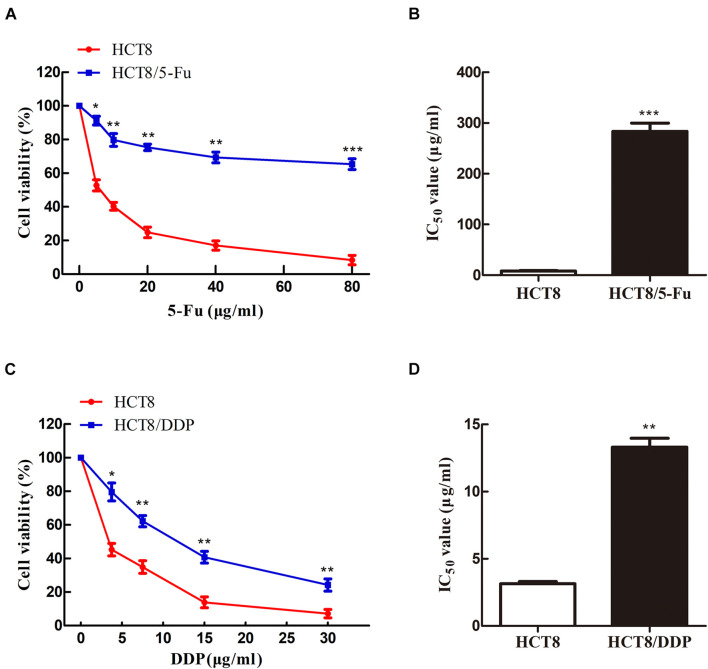
HCT8/5-Fu and HCT8/DDP cells were more resistant to chemotherapy drugs. Cell viability and IC_50_ of HCT8 and HCT8/5-Fu cells **(A,B)**, HCT8, and HCT8/DDP **(C,D)** were assessed by MTT assay. *p < 0.05; **p < 0.01 and ***p < 0.001.

To obtain the chemoresistant CRC cell lines (HCT8/5-Fu and HCT8/DDP), the parental cells HCT8 were exposed to increasing concentrations of 5-Fu or cisplatin for more than 7 months. MTT experiments were used to detect the sensitivity of HCT8 and HCT8/5-Fu cells to 5-Fu, as well as HCT8 and HCT8/DDP cells to cisplatin. The results showed that the cell viability of all groups decreased with the concentration of the drug increased. However, the survival rate of HCT8 cells was significantly lower than that of HCT8/5-Fu or HCT8/DDP cells when the same concentration was add (Figures 1A,C). Moreover, the half maximal inhibitory concentration (IC_50_) values of HCT8/5-Fu and HCT8/DDP were higher than those of chemosensitive cell lines (Figures 1B,D), indicating that the two drug-resistant cell lines were more resistant to drugs than paternal cell lines, which lays the foundation for follow-up research.

### Expression Profiles of Circular RNAs

**FIGURE 2:**
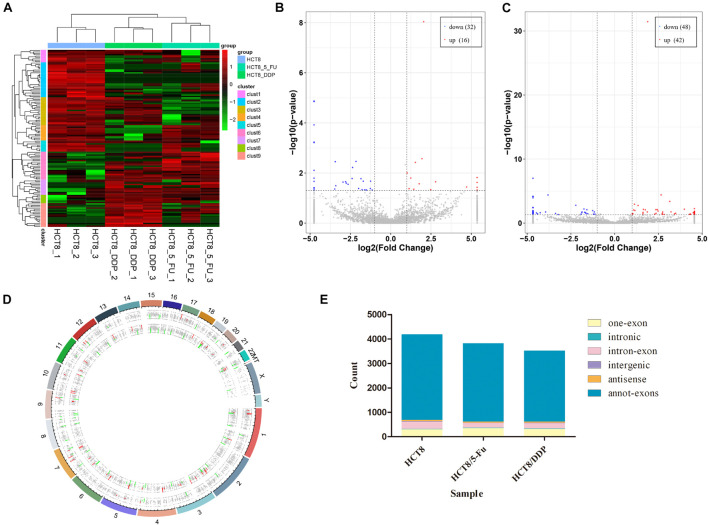
Expression profiles of circRNAs. **(A)** Cluster analysis of DE-circRNAs. The volcano plot of DE-circRNAs in **(B)** HCT8/5-Fu cell lines and **(C)** HCT8/DDP cell lines. Red denotes increased expression, and blue indicates decreased expression. **(D)** Distribution of circRNAs on different chromosomes. **(E)** Genomic origin of the identified circRNAs.

The circRNA expression profiles of the parental cell lines and two drug-resistant cell lines were obtained through RNA-sequencing. DE-circRNAs were screened out in drug-resistant cells compared to parental cells with the criteria of |log2FoldChange| > 1 and *p* < 0.05. The heat map analysis showed the expression of the DE-circRNAs visually; the three repeats of each group clustered together, while the chemoresistant groups and control group were clustered separately (Figure 2A). The sequencing results indicated that a total of 7,393 circRNAs were screened out in HCT8/5-Fu cells, among which 48 circRNAs were DE, with 16 upregulated and 32 downregulated (Figure 2B). The top five up and downregulated circRNAs in HCT8/5-Fu cell lines are shown in Supplementary Table 1. In addition, 90 DE-circRNAs (42 upregulation and 48 downregulation) were found among 7,385 circRNAs in HCT8/DDP cells (Figure 2C). The top five up and downregulated circRNAs in HCT8/DDP cell lines are shown in Supplementary Table 2. In addition, the distribution of circRNAs on different chromosomes was visualized (Figure 2D). As shown in Figure 2E, the majority of candidate circRNAs originate from the exonic regions, and the rest were introns, antisense, and intergenic.

### Gene Ontology Analysis of DE-Circular RNAs

**FIGURE 3:**
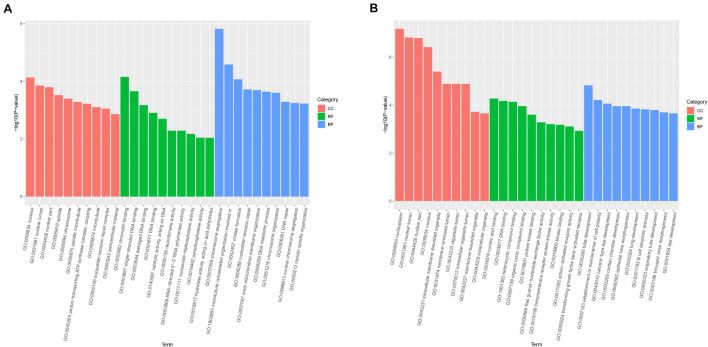
Gene Ontology analysis of host genes of DE-circRNAs. GO analysis of the DE-circRNAs in **(A)** HCT8/5-Fu cell lines and **(B)** HCT8/DDP cell lines.

Gene Ontology enrichment analysis was mainly classified to molecular function (MF), BP, and cell component (CC). The host genes of the DE-circRNAs in HCT8/5-Fu cells have a total of 1,887 GO functional annotations, among them, 1,369, 256, and 262 GO terms were significantly enriched in BP, CC, and MF, respectively (Figure 3A). The GO analysis of HCT8/DDP cells showed that these host genes of the DE-circRNAs were enriched to 3,208 GO entries, which were mainly related to tube development (BP), nucleoplasm and nuclear cavity (CC), and nucleic acid binding and DNA binding (MF), as shown in Figure 3B.

### Kyoto Encyclopedia of Genes and Genomes Pathway Analysis of DE-Circular RNAs

**FIGURE 4:**
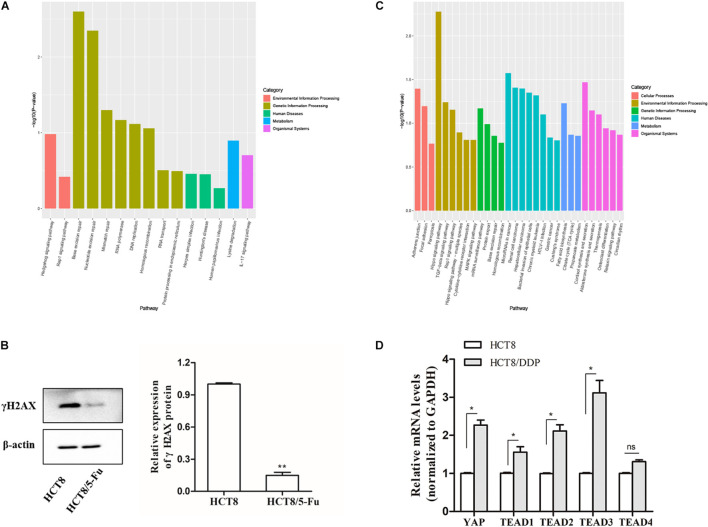
Kyoto Encyclopedia of Genes and Genomes enrichment analysis of host genes of DE-circRNAs. **(A)** Significantly enriched signaling pathways of the DE-circRNAs in HCT8/5-Fu cell lines. **(B)** The expression of γH2AX in HCT8 and HCT8/5-Fu cells was detected *via* Western blotting. **(C)** Significantly enriched signaling pathways of the DE-circRNAs in HCT8/DDP cell lines. **(D)** The expression of key genes of Hippo signaling in HCT8 and HCT8/DDP cells was detected by qRT-PCR. *n* = 3, data are shown as mean ± SD, *p < 0.05; **p < 0.01 and “ns” represents no statistical significance.

Kyoto Encyclopedia of Genes and Genomes pathway enrichment analysis showed that the host genes of the DE-circRNAs in HCT8/5-Fu cells were enriched in 15 pathways, of which base excision repair, nucleotide excision repair, and mismatch repair were highly enriched, indicating the development of 5-Fu resistance in CRC cells was possibly related to the DNA repair pathway, as shown in Figure 4A. γH2AX, a biomarker of DNA damage, was detected by Western blotting; the expression level of γH2AX in parental cell lines was higher than that of HCT8/5-Fu cells, suggesting HCT8/5-Fu cells have stronger DNA damage repair capacity (Figure 4B). In addition, the KEGG pathway enrichment analysis of the DE-circRNAs in HCT8/DDP cells showed that the Hippo signaling pathway was the most enriched pathway (Figure 4C). Then, the key genes of Hippo signaling were detected *via* qRT-PCR; the expression level of YAP1 and TEAD family transcription factors (except TEAD4) was significantly highly expressed in HCT8/DDP cells compared to that of parental cell lines, indicating inactivation of the Hippo signaling may play an important role in the development of cisplatin resistance (Figure 4D), which was consistent with previous studies on the involvement of the Hippo signaling pathway in multiple cancer chemoresistance (Wang et al., 2016, 2018).

### Validation of Common DE-Circular RNAs by Quantitative Real-Time PCR

**FIGURE 5:**
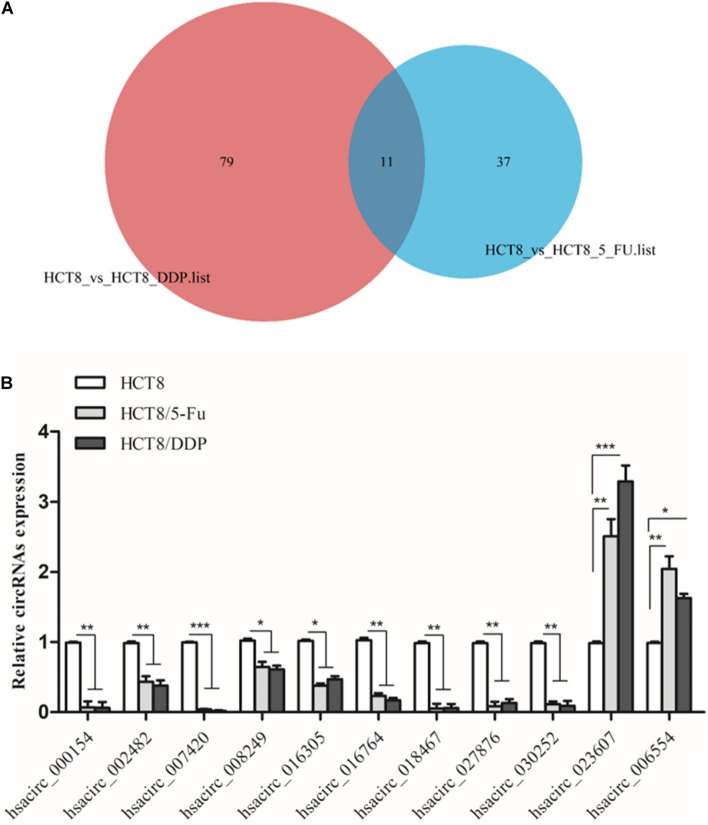
Screening and verification of common DE-circRNAs. **(A)** Venn diagram displays DE- and overlapping circRNAs between HCT8/5-Fu and HCT8/DDP cell lines. **(B)** The relative expression levels of common DE-circRNAs in chemoresistant and chemosensitive cell lines were determined by qRT-PCR. *n* = 3, data are shown as mean ± SD, *p < 0.05; **p < 0.01 and ***p < 0.001.

Venn analysis was used to screen common DE-circRNAs in two drug-resistant cells, as shown in Figure 5A; 11 common DE-circRNAs were found, with 2 upregulation and 9 downregulation (Table 2). qRT-PCR was used to detect the expression levels of common DE-circRNAs to verify the accuracy of RNA-sequencing. The verification results were consistent with the sequencing data, indicating the high accuracy of RNA-sequencing results. Among them, hsacirc_023607 was the most upregulated DE-circRNAs, about three times upregulated; and hsacirc_007420 was the most downregulated DE-circRNAs, which was expressed extremely low in CRC chemoresistant cell lines (Figure 5B).

**TABLE 2:** Common DE-circRNAs in chemoresistant cell lines.

Gene ID	HCT8/5-Fu			HCT8/DDP		
	Regulation	log_2_FC	*p*-Value	Regulation	log_2_FC	*p*-Value
hsacirc_023607	Up	2.0468	9.16227E−09	Up	1.8896	3.58054E−32
hsacirc_006554	Up	1.5311	0.003791891	Up	1.0002	0.036777448
hsacirc_007420	Down	−Inf	0.000120304	Down	−Inf	7.14213E−05
hsacirc_027876	Down	−Inf	1.40889E−05	Down	−Inf	9.98353E−08
hsacirc_018467	Down	−Inf	0.000582425	Down	−Inf	6.60779E−05
hsacirc_000154	Down	−Inf	0.038921704	Down	−Inf	0.012607355
hsacirc_030252	Down	−Inf	1.40889E−05	Down	−3.8073	4.04837E−05
hsacirc_016305	Down	−3.4734	0.043758497	Down	−Inf	0.00402312
hsacirc_016764	Down	−1.6087	0.046626401	Down	−1.5865	0.008076318
hsacirc_002482	Down	−1.2670	0.041782606	Down	−1.1604	0.018296181
hsacirc_008249	Down	−1.2489	0.021168291	Down	−1.2083	0.039039397

*FC, fold change; Inf, infinity.*

### Prediction of Circular RNA–miRNA Network

**FIGURE 6:**
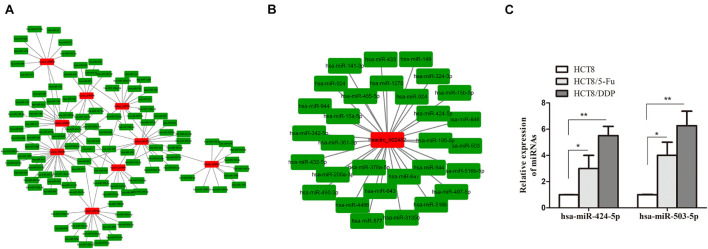
Prediction of circRNA–miRNA regulatory networks. **(A)** The network of targeting miRNAs of common DE-circRNA related to cancer resistance to 5-Fu or cisplatin. **(B)** The regulatory network of the hub gene hsacirc_002482 targeted miRNAs related to 5-Fu or cisplatin resistance. **(C)** The expression levels of hsa-miR-424-5p and hsa-miR-503-5p in chemoresistant and chemosensitive cell lines were determined by qRT-PCR. *n* = 3, data are shown as mean ± SD, *p < 0.05 and **p < 0.01.

Circular RNAs exist as an miRNA response element (MRE) and could act as “molecular sponges” of miRNAs, inhibiting its expression. miRanda and circinteractome online tools were used to predict the targeting miRNAs of common DE-circRNA in CRC chemoresistance cell lines. A total of 951 miRNAs were predicted and displayed (Supplementary Figure 1A), of which the most upregulated circRNA (hsacirc_023607) and downregulated circRNA (hsacirc_007420) targeted 110 and 253 miRNAs, respectively (Supplementary Figures 1B,C). In order to explore whether hsacirc_023607 affects CRC chemoresistance, we knocked down the expression of hsacirc_023607 in two chemoresistant cells. As shown in Supplementary Figure 2A, the siRNAs significantly decreased hsacirc_023607 expression level. Unfortunately, we found silencing hsacirc_023607 has no effect on chemosensitivity, cell apoptosis, and proliferation (Supplementary Figures 2B–D). Therefore, we speculated that the DE-circRNAs obtained by RNA-sequencing are not all related to chemoresistance; subsequently, we further screened 133 targeting miRNAs of common DE-circRNA related to cancer resistance to 5-Fu or cisplatin (Figure 6A). Among which, hsacirc_002482 was identified as the hub gene through the circRNA–miRNA network analysis; hence, we selected hsacirc_002482 as a candidate circRNA for further investigation. The network of hsacirc_002482 targeted 29 chemoresistance-related miRNAs was constructed (Figure 6B), among which hsa-miR-424-5p and hsa-miR-503-5p have been reported to regulate cisplatin and 5-Fu resistance and related to CRC chemosensitivity (Qiu et al., 2013; Xu et al., 2017; Yu et al., 2020). Thus, we detected the expression of hsa-miR-424-5p and hsa-miR-503-5p in parental and chemoresistant cell lines and found all of them were significantly highly expressed in chemoresistant cell lines (Figure 6C); hence, we also speculate that hsacirc_002482 might play an important role in CRC chemoresistance.

### Overexpression of hsacirc_002482 Enhances Colorectal Cancer Chemosensitivity

**FIGURE 7:**
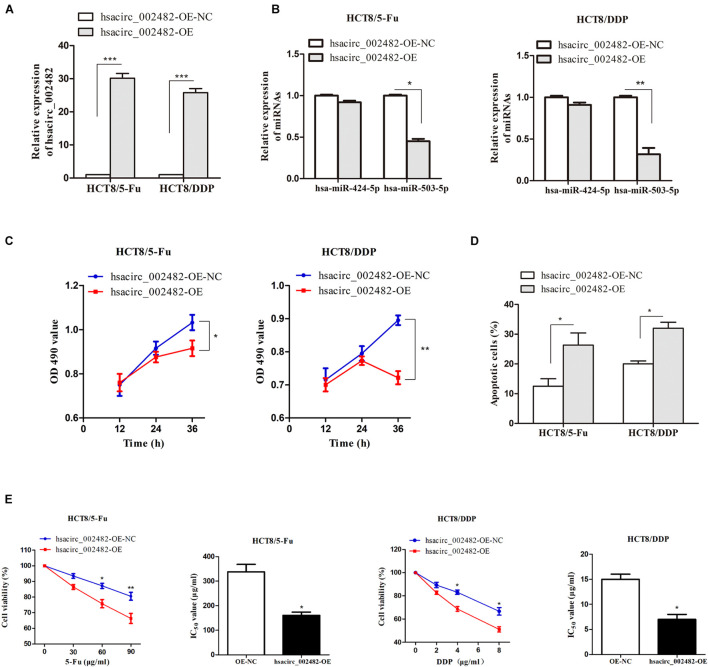
Overexpression of hsacirc_002482 sensitizes CRC chemoresistant cells to 5-Fu and cisplatin. The expression of **(A)** hsacirc_002482 and **(B)** hsa-miR-424-5p and hsa-miR-503-5p in chemoresistant cells after hsacirc_002482 transfection. Overexpressing hsacirc_002482 **(C)** inhibited proliferation, **(D)** induced apoptosis, and **(E)** enhanced chemosensitivity of chemoresistant cells after treatment with chemotherapeutic drugs. *n* = 3, data are shown as mean ± SD, *p < 0.05, **p < 0.01 and ***p < 0.001.

To further explore the functional roles of hsacirc_002482 in CRC chemoresistance, we first overexpressed the hsacirc_002482 in HCT8/5-Fu and HCT8/DDP cell lines (Figure 7A). Then, we found elevated hsacirc_002482 partially reduced the expression of hsa-miR-503-5p, but not hsa-miR-424-5p (Figure 7B). Subsequently, cell proliferation, apoptosis, and viability were examined after treatment with chemotherapeutic drugs. The results showed that hsacirc_002482 upregulation could inhibit cell proliferation as well as increase apoptosis ratios of two CRC chemoresistant cells (Figures 7C,D). Additionally, overexpression of hsacirc_002482 could significantly enhance chemosensitivity of HCT8/5-Fu and HCT8/DDP cells compared to negative control, and the IC_50_ values were decreased (Figure 7E), indicating hsacirc_002482 was associated with 5-Fu and cisplatin resistance in CRC.

## Discussion

Chemotherapy is an important treatment method for CRC patients and widely used to prolong the survival time of patients (Ping et al., 2018; Vodenkova et al., 2020). 5-Fu and cisplatin are common chemotherapeutic drugs; however, the development of chemoresistance limited the therapeutic effect and resulted in poor prognosis (Ye et al., 2019). Therefore, exploring the mechanisms of chemoresistance in CRC is particularly important. CircRNAs are a new type non-coding RNAs, regarded as stable transcription products and potential prognosis biomarkers for cancers (Qu et al., 2015; Zhang et al., 2018; Arnaiz et al., 2019). Numerous studies have found that circRNAs participate in several biological functions, and dysregulated circRNAs are closely related to cell proliferation, migration, invasion, and chemoresistance of cancers (Li et al., 2018; Abu et al., 2019; Wang et al., 2019). For example, hsa_circ_0000745 was confirmed as a tumor promoter in cervical cancer progression; its overexpression promoted cell proliferation, migration, and invasion, which regarded as a prognosis marker of cervical cancer (Jiao et al., 2020). Jian et al. (2020) indicated hsa_circ_001680 was highly expressed in CRC tissue, which could enhance the capacity of cell proliferation and migration. Additionally, hsa_circ_001680 promoted the cancer stem cell population and induced irinotecan resistance by regulating miR-340 to affect BMI1 expression (Jian et al., 2020). Until now, the expression profiles and functions of circRNAs in CRC chemoresistance remain largely unknown; hence, the study aims to find potential circRNAs that regulate 5-Fu and cisplatin resistance in CRC.

The development of high-throughput sequencing technology provides the possibility to study circRNAs. In the present study, the expression profiles of circRNAs were compared between parental cells (HCT8) and two drug-resistant cell lines (HCT8/5-Fu and HCT8/DDP) through RNA-sequencing. The results demonstrated 48 circRNAs were aberrantly regulated in HCT8/5-Fu cells, and 90 circRNAs with significant differences were detected in HCT8/DDP cells. Subsequently, a total of 11 common DE-circRNAs in HCT8/5-Fu and HCT8/DDP cells were identified.

Gene Ontology analysis was performed to analyze the function of the host genes of dysregulated circRNAs; previous studies have reported that many factors were closely related to chemoresistance, such as mitochondria (Lee et al., 2021), nucleotide excision repair (Duan et al., 2020), DNA repair (Colomer et al., 2019), and cell apoptotic process (O’Connell et al., 2021), which were also confirmed in this study. In addition, KEGG pathway analysis was used to determine the main biochemical metabolic pathways and signaling pathways of DE-circRNAs. The DE-circRNAs in HCT8/5-Fu cells were mainly enriched in DNA repair pathways, including base excision repair, nucleotide excision repair, and mismatch repair. Accumulating evidence demonstrated that DNA repairs were significantly associated with chemoresistance of cancer. Sakthivel and Hariharan (2017) showed that the activation of various oncogenes, cancer stem cells, transcription factors, signaling pathways, and low oxygen environment in cancer cells could effectively repair DNA damage, resulting in repaired cancer cells getting more resistant to chemotherapy. Meng et al. (2015) also reported that abnormal activation of the Hedgehog signaling pathway in tumors can cause chemoresistance through the DNA repair process. In addition, several studies have shown that the Hippo signaling pathway participated in chemoresistance of various cancers, such as osteosarcoma (Wang et al., 2016), bladder cancer (Xia et al., 2018), ovarian cancer (Xu et al., 2018), CRC (Wang et al., 2018), and breast cancer (Khanal et al., 2019). In the study, KEGG pathway analysis in HCT8/DDP cells showed that DE-circRNAs may affect many pathways such as Hippo, transforming growth factor-β (TGF-β), and mitogen-activated protein kinase (MAPK) signaling pathway, among them Hippo signaling pathway was the most enriched pathways, which may be related to the process of cisplatin resistance in CRC.

The expression levels of the common DE-circRNAs were confirmed using qRT-PCR, which were well consistent with the RNA-sequencing data. Additionally, compared with parental cells, hsacirc_023607 (upregulation) and hsacirc_007420 (downregulation) were found to have the largest expression fold change in chemoresistant cell lines. It has been reported that circRNAs could serve as sponges of miRNAs to regulate its expression (Thomson and Dinger, 2016; Zhong et al., 2018; Lai et al., 2020). A recent study showed that circCRIM1 prevented its inhibitory effect on the target gene FOXQ1 through competitive binding with miR-422a, promoted the metastasis of nasopharyngeal carcinoma, and developed resistance to docetaxel chemotherapy (Hong et al., 2020). In addition, Zhan et al. confirmed that hsa_circRNA_103809 can regulate the resistance to cisplatin of non-small cell lung cancer through the miR-377-3p/GOT1 axis *in vivo* and *in vitro* (Zhan et al., 2020). In the study, 951 miRNAs were predicted as target genes of common DE-circRNAs by miRanda and circinteractome database; we first chose hsacirc_023607 with the highest upregulation to study its correlation with drug resistance. However, silencing hsacirc_023607 does not affect CRC chemoresistance, indicating not all common DE-circRNAs related to drug resistance; further research is needed. Moreover, we filtered 133 targeted miRNAs related to cancer 5-Fu or cisplatin-resistance from 951 miRNAs. hsacirc_002482 was decreased in two drug-resistant cells and identified as the hub gene in the circRNA–miRNA network; hence, we selected it for further research. We found 29 miRNAs related to cancer resistance to 5-Fu or cisplatin were targeted by hsacirc_002482, in which hsa-miR-424-5p and hsa-miR-503-5p have been reported to regulate chemosensitivity of cisplatin and 5-Fu and also related to CRC resistance (Yang et al., 2017; Liu et al., 2021). In addition, the expressions of hsa-miR-424-5p and hsa-miR-503-5p were detected by qRT-PCR, and both were highly expressed in chemoresistance cell lines. To further investigate the biological functions of hsacirc_002482 in CRC chemoresistance, gain-of-function assays were conducted. We first detected the expression level of hsa-miR-503-5p and hsa-miR-424-5p and found hsacirc_002482 overexpression reduced the expression of hsa-miR-503-5p instead of hsa-miR-424-5p. Additionally, we also found hsacirc_002482 upregulation not only inhibited cell proliferation and promoted cell apoptosis but also significantly enhanced chemosensitivity of HCT8/5-Fu and HCT8/DDP cells. Thus, we speculated that the hsacirc_002482/hsa-miR-503-5p axis may play important roles in CRC chemoresistance.

In general, the study screened circRNAs profiles in chemosensitive and resistant CRC cell lines by RNA-sequencing. The common DE-circRNAs in HCT8/5-Fu and HCT8/DDP cells were screened out, and their expressions were validated by qRT-PCR. hsacirc_023607 and hsacirc_007420 were identified as the highest upregulation and downregulation circRNAs, respectively. Targeted miRNAs of common DE-circRNAs were predicted by bioinformatics methods. However, functional studies showed hsacirc_023607 has no effect on CRC chemoresistance. Thus, the networks of miRNAs related to cancer resistance to 5-Fu or cisplatin were constructed, in which hsacirc_002482 was regarded as the hub gene. Moreover, hsacirc_002482 overexpression could increase the chemosensitivity of HCT8/5-Fu and HCT8/DDP cells, suggesting hsacirc_002482 may play important roles in the development of CRC chemoresistance. However, further in-depth functional and mechanistic studies of hsacirc_002482 are required, which we will continue to undertake in the future.

## Data Availability Statement

We have uploaded our data to the GEO database (GSE173606).

## Author Contributions

QWu, FY, QWa, XX, and CZ designed the experiments. QWu, FY, and XX analyzed the RNA-sequencing data. FY, CZ, and XH performed the experiments. XX, QH, and ZX performed statistical analysis. All authors wrote the manuscript and reviewed drafts, and agreed with its submission.

## Conflict of Interest

The authors declare that the research was conducted in the absence of any commercial or financial relationships that could be construed as a potential conflict of interest.

## Publisher’s Note

All claims expressed in this article are solely those of the authors and do not necessarily represent those of their affiliated organizations, or those of the publisher, the editors and the reviewers. Any product that may be evaluated in this article, or claim that may be made by its manufacturer, is not guaranteed or endorsed by the publisher.

## Footnotes

1
http://ccb.jhu.edu/software/hisat2/index.shtml

2
http://geneontology.org/

3
http://www.kegg.jp/
