# Supplementary material for: Identification of Circular RNAs Associated With Chemoresistance in Colorectal Cancer
Source: Front Genet. 2021 Sep 17;12:696948. doi: 10.3389/fgene.2021.696948 (PMC8484910; doi:10.3389/fgene.2021.696948)
Supplement: Supplementary file 1 [file Data_Sheet_1.docx]

Supplementary Material

# Supplementary Tables

**Table S1**The top five up and down-regulated circRNAs inHCT8/5-Fu cell lines

| Gene ID | HCT8/5-Fu | | | |
| --- | --- | --- | --- | --- |
|  | regulation | log_2_FC | *p* value | Source Gene |
| hsacirc_000579 | up | Inf | 0.015 | POLR2A |
| hsacirc_019817 | up | Inf | 0.0238 | KRIT1 |
| hsacirc_018210 | up | Inf | 0.0372 | RAD23B |
| hsacirc_012514 | up | Inf | 0.048 | RCC1 |
| hsacirc_018494 | up | Inf | 0.0492 | AUTS2 |
| hsacirc_007420 | down | -Inf | 0.0001 | NUSAP1 |
| hsacirc_030252 | down | -Inf | 0.00001 | DMC1 |
| hsacirc_027876 | down | -Inf | 0.00001 | GRHL2 |
| hsacirc_018467 | down | -Inf | 0.0005 | CCDC91 |
| hsacirc_010980 | down | -Inf | 0.0078 | MT-ATP8 |

**Table S2** The top five up and down-regulated circRNAs inHCT8/DDP cell lines

| Gene ID | HCT8/DDP | | | |
| --- | --- | --- | --- | --- |
|  | regulation | log_2_FC | *p* value | Source Gene |
| hsacirc_022191 | up | Inf | 0.0054 | CREB1 |
| hsacirc_020305 | up | Inf | 0.0142 | FOXP1 |
| hsacirc_021186 | up | Inf | 0.016 | FIP1L1 |
| hsacirc_027418 | up | Inf | 0.0197 | DHTKD1 |
| hsacirc_021191 | up | Inf | 0.0226 | LARP4B |
| hsacirc_027876 | down | -Inf | 0.00036 | GRHL2 |
| hsacirc_018467 | down | -Inf | 0.00004 | CCDC91 |
| hsacirc_007420 | down | -Inf | 0.00007 | NUSAP1 |
| hsacirc_029352 | down | -Inf | 0.0001 | PPM1D |
| hsacirc_016305 | down | -Inf | 0.004 | TMEM192 |

## Supplementary Figure


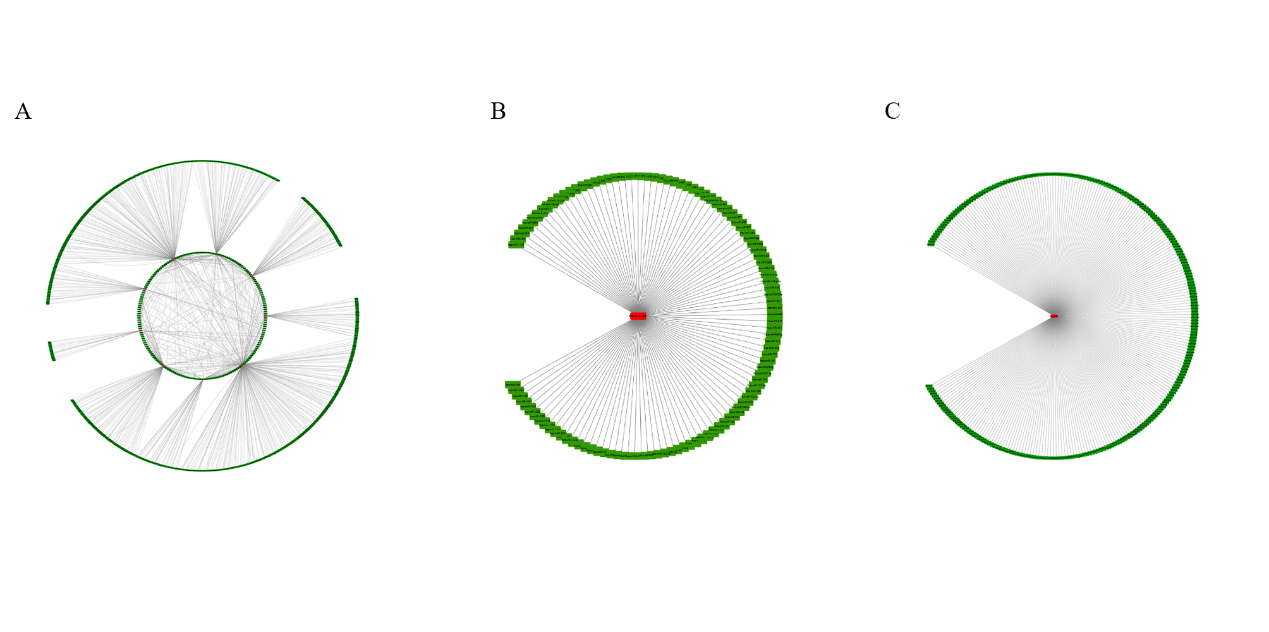


**Figure S1** Prediction of circRNA/miRNA networks. **(A)** The prediction network of miRNAs targeted by common DE-circRNAs. **(B)** The regulatory network of hsacirc_023607. **(C)** The regulatory network of hsacirc_007420.Red denoted circRNAs, and green indicated miRNAs.


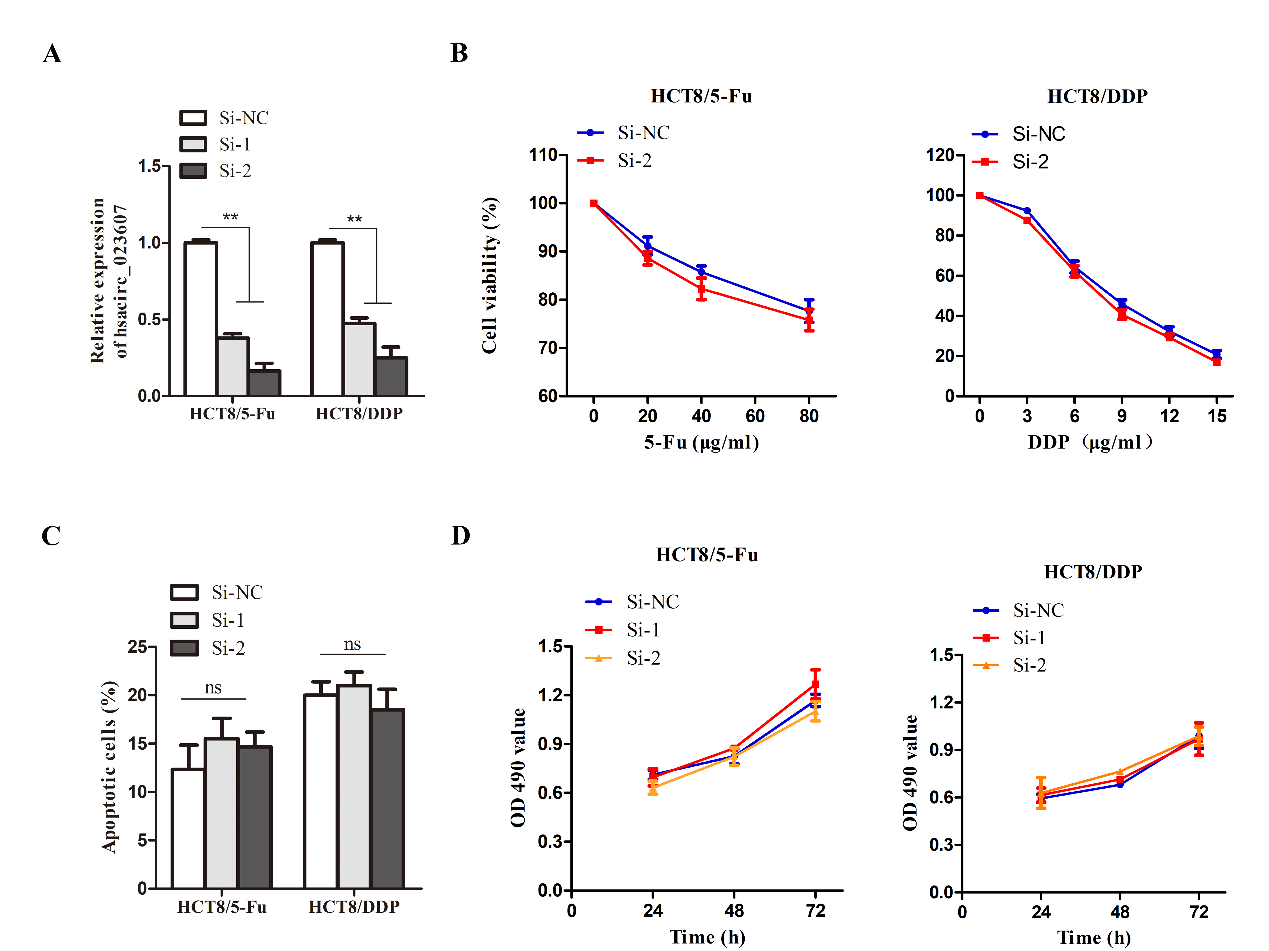


**Figure S2** The effects of silencing hsacirc_023607 on chemoresistance of CRC. (A) The expression of hsacirc_023607 in chemo-resistant cells after hsacirc_023607 transfection. Silencing hsacirc_002482 have no effect on **(B)** chemosensitivity, **(C)**cell apoptosis, **(D)** cell proliferation of chemo-resistant cells after treated with chemotherapeutic drugs. *n* = 3, data were shown as mean ± SD, ** *p* <0.01,“ns” represented nostatistical significance.
